# Supplementary material for: Conformational free-energy landscapes of a Na+/Ca2+ exchanger explain its alternating-access mechanism and functional specificity
Source: Proc Natl Acad Sci U S A. 2024 Apr 8;121(16):e2318009121. doi: 10.1073/pnas.2318009121 (PMC11032461; doi:10.1073/pnas.2318009121)
Supplement: Supplementary file 1 — Appendix 01 (PDF) [file pnas.2318009121.sapp.pdf]

Supporting Information

Conformational free-energy landscapes of a Na^+/Ca^{2+} exchanger explain its alternating-access mechanism and functional specificity

Fabrizio Marinelli[1]* and José D. Faraldo-Gómez [1]*

[1]Theoretical Molecular Biophysics Laboratory
National Heart, Lung and Blood Institute
National Institutes of Health, Bethesda, MD 20814

*Correspondence should be addressed to:
José D. Faraldo-Gómez (jose.faraldo@nih.gov) or
Fabrizio Marinelli (fabrizio.marinelli@nih.gov)

March 1st, 2024

Supporting Movies

Movie S1. Alternating-access mechanism of NCX_Mj bound to 3 Na^+ ions, as predicted by enhanced-sampling all-atom MD simulations and minimum free-energy path calculations. Helices TM1 and TM6 are highlighted in purple, and water molecules in the proximity of the ion binding sites are shown as red spheres; the bound Na^+ ions are shown in green. All other elements of the simulation system are omitted for clarity.

Movies S2. Alternating-access mechanism of NCX_Mj bound to 1 Ca^{2+} ions, as predicted by enhanced-sampling all-atom MD simulations and minimum free-energy path calculations. Helices TM1 and TM6 are highlighted in purple, and water molecules in the proximity of the ion binding sites are shown as red spheres; the bound Ca^{2+} ion is shown in cyan. All other elements of the simulation system are omitted for clarity.

Supporting Methods

Construction and simulation of repeat-swap model of NCX_Mj – To construct the repeat-swap model of NCX_Mj shown in Fig. 1, we first generated a reference sequence by swapping the residues of the two repeats, with the specific sequence alignment provided in Fig. S1, as described elsewhere (1). We then generated 250 models with MODELLER v9.15 (2) using as template the OF X-ray structure of NCX_Mj bound to 3 Na^+ ions (3). To preserve the C_2 pseudo-symmetry of the binding site region with respect to an axis parallel to the membrane plane, we restrained the distances between the 3 Na^+ ions and their coordinating oxygens as well as the oxygen-oxygen distances. The lowest-energy model was selected for further refinement. First, sidechain rotamers were optimized using SCWRL4 (4). Then, the model was energy-minimized according to the CHARMM27/CMAP forcefield (5, 6) and embedded in a solvated POPC bilayer using GRIFFIN (7). An MD simulation of 800 ns was then carried out to equilibrate the model at constant temperature (298 K), pressure (1 atm) and membrane surface area (~69 Å^2 per lipid). This simulation was carried out with NAMD 2.12 (8) and the same force field. This initial equilibration involved a series of stages in which first position restraints and then RMSD restraints are gradually removed on protein sidechain and backbone atoms. Thereafter we performed additional 3-μs MD simulation without any restraint (see Fig. S5C for results).

Improved refinement of crystallographic data for NCX_Mj bound to Ca^{2+} and Sr^{2+} – A statistical survey of the Ca^{2+}-binding sites in the PDB (9) suggests that when Ca^{2+} is bound to two carboxylate groups in a bidentate mode, the latter ion has a total coordination of eight. To fulfill this coordination geometry, in our previous simulation study of NCX_Mj, based on the OF X-ray structure (9), we placed Ca^{2+} in the S_{Ca} site and introduced two water molecules coordinating Ca^{2+} at near the S_{mid} site. This ion coordination mode has also been used in this study. By contrast, in the original interpretation of the crystallographic data, Ca^{2+} was assumed to partially occupy S_{Ca} and S_{mid} sites, assisted by one water molecule (9). To validate or refute our previous interpretation, we carried out a new refinement of NCX_Mj bound to Ca^{2+} based on the electron-density map available in the Protein Data Bank (entry 5HXR). Model building was completed with COOT (10), and structure refinement was carried out with PHENIX (11). The results are shown in Fig. S2. It is clear that the geometry we propose for the Ca^{2+} state is just as compatible with the electron-density data as the original model; it is however more plausible, statistically speaking, and is also supported by recent ATR-FTIR experiments (12), which show that Ca^{2+} does not reside near residue D240 at the S_{mid} site. To further support the proposed coordination geometry, we also carried out a new refinement of crystallographic data obtained for Sr^{2+} (9), which is also transported by NCX_Mj (13). This analysis clearly reveals that in the published structural model (entry 5HXS) the coordination of the Sr^{2+} ion is incomplete since additional coordinating atoms ought to be present near the S_{mid} site (Fig. S2). Indeed, a new refinement adding two coordinating

water molecules in the defective region significantly improves the agreement with the crystallographic data (Fig. S2). The conclusion from this analysis is that both Ca^{2+} and Sr^{2+} bind only at the S_{Ca} site and in a nearly identical mode, which includes two coordinating water molecules at the S_{mid} site. The newly refined structures of NCX_Mj bound to Ca^{2+} or Sr^{2+} are available at https://github.com/Faraldo-Gomez-Lab-at-NIH/Download.

Enhanced-sampling simulations of NCX_Mj bound to 3 Na^+ or 1 Ca^{2+} – The enhanced-sampling MD simulations used to calculate the free-energy landscapes shown in Fig. 2 used as only input the outward-facing, occluded structure of NCX_Mj with 3 Na^+ ions bound; the protein coordinates are those initially reported (PDB entry 3V5U (3), but the ion-binding site configuration reflects a subsequent correction (14)). A total of 18 reaction coordinates were used simultaneously in these calculations; collectively, these coordinates were designed to loosely guide the simulated trajectories to sample the vicinity of an idealized linear path between the OF structure and the repeat-swap (RS) model of the IF state. The functional form of each of these variables is:

$$\xi_i(t) = \frac{exp\{-\lambda d_1(t)\} + 2exp\{-\lambda d_2(t)\}}{exp\{-\lambda d_1(t)\} + exp\{-\lambda d_2(t)\}} \qquad (1)$$

where $d_1(t)$, $d_2(t)$ quantify the similarity of the configuration at simulation time t relative to either of the two reference structures. (Note that neither was assumed to be a free-energy minimum.) The smoothing parameter λ is proportional to the inverse of d_{12} i.e. the similarly between the two end states (the values λ for each variable are given in Table S1). The particular metric of similarity used in Eq. 1 is the mean-square difference (MSD) in the configuration of a specific atom selection. Each of the 18 different coordinates ξ_i was defined using a different atom selection. In each case, configurations close to the experimental OF structure correspond to ξ_i~1, while those close to the RS model correspond to ξ_i~2. All other configurations result in intermediate values of ξ_i. The specific atom selections that define each of the 18 path-coordinates ξ_i are graphically depicted in Fig. S3. These selections include backbone and sidechains atoms, in both structured and unstructured fragments, both on the intracellular and the extracellular side of the protein; altogether, they describe global and local structural differences between the internal structures of the two topological repeats with the structure of the OF state (Fig. 1). Preliminary non-equilibrium simulations of the alternating-access transition using this set of path-coordinates and the minimum-mode Metadynamics method (18) indicated this selection was suitable for the problem at hand.

It is important to note, however, that this selection did not result from an automated process or blind algorithm; such methodology would be very valuable, but to our knowledge it does not exist at present time. Instead, this selection was based on our accumulated experience (i.e. both successes and failures) evaluating conformational free-energy landscapes of proteins

through enhanced-sampling simulation methods at quasi-equilibrium (9, 15-17). This experience tells us that to induce a conformational change in simulation, one must act on the elements that govern the energetics of the molecular system. Simple descriptors of the conformational state of a protein might appear intuitive but are oftentimes lacking in this regard. The backbone of a protein, for example, is a good descriptor for a graphical representation of a structure, but in reality, the tertiary structure of the protein is largely dictated by the interactions between sidechains and their environment. In particular, the re-organization of hydrophobic sidechains into alternate interaction patterns is typically a rate-limiting process that must be explicitly considered; rearrangements of sidechains in seemingly unstructured elements are also slow processes that can be highly consequential.

The BE-META simulations carried out to explore this 18-dimensional space included 32 replicas. In the simplest formulation of the BE-META method, each of the replicas includes a Metadynamics bias that fosters exploration of one reaction coordinate. In previous studies (16, 17) we have observed that the performance of the method is much improved when each replica is designed to explore at least two coordinates, in different combinations (detailed in Table S1). To include a replica for which the sampling is totally unbiased also provides a useful metric of self-consistency. Monte-Carlo exchanges between replicas were attempted every 10-20 ps.

For state with 3 Na^+ ions bound, we carried out an initial equilibration of 1 μs for each replica; in this time window, all replicas reached uniform sampling in the corresponding reaction-coordinate space, indicating that the bias-potential developed in each replica approximately mirror the corresponding free-energy profile (or potentials-of-mean-force). We also observed that 7 trajectories visited independently an IF state distinct from the RS model. This state was also well populated in the unbiased replica, confirming that it is a low free-energy minimum. To initiate the calculations with 1 Ca^{2+} ion bound, we extracted the final configurations from each of the replicas used for the Na^+-bound state, and in each case, we reconstructed the binding site geometry of Ca^{2+}-bound state (Fig. S2) through a series of restrained equilibrations totaling 50 ns, while sustaining the biasing potential previously developed in each replica. To fully converge the Metadynamics biasing potentials, we then duplicated the number of replicas to 64 and extended the sampling by 200 ns per replica for the Na^+ state and by 250 ns per replica for the Ca^{2+} state. The free-energy landscapes shown in Fig. 2 derive from this 64-replica calculation, for the final 200-ns sampling window.

Enhanced-sampling simulations of NCX_Mj bound to 2 H^+, 2 Na^+ or with no ions bound – Our previous simulation studies of OF NCX_Mj bound to 2 H^+, 2 Na^+ or with no ions bound indicated that the alternating-access transition would be limited by the energetics of occlusion of the binding-site region (9), rather than a conformational intermediate between the OF and IF state. Thus, the enhanced-sampling simulations used to derive the free-energy landscapes in Fig. 5 focused instead on fostering the occlusion or opening of the binding site, by using an alternative

set of reaction coordinates. The first type of coordinate quantifies the degree of hydration of each of the ion binding sites. For example, for the central site, known as S_{Ca}, this reaction coordinate is:

$$\xi_{Ca}(t) = \frac{\beta}{ln \sum_j exp\{\beta / r_j(t)\}} \tag{2}$$

where $r_j(t)$ denotes the time-dependent distance between the center of the site and each of the oxygen atoms in any of the water molecules in the system, denoted by the index j; β is 10 nm. When the binding site is occupied by an ion, the ion defines its center; when the site is empty, its center is defined by the center of mass of the protein oxygen atoms coordinating the ion when bound. Analogous expressions apply to the other binding sites (except for Glu residues, for which the C_δ atom was considered instead). The second type of reaction coordinate describes instead changes in the protein structure. For example, in the examination of the IF states, $\xi_{TM1,2-7}$ describes the movement of intracellular portions of TM1 (residues 18-26) and TM2 (residues 45-50) relative to TM7 (residues 211-217), using the same function as that in Eq. 1:

$$\xi_{TM1,2-7}(t) = \frac{exp\{-\lambda d_1(t)\} + 2exp\{-\lambda d_2(t)\}}{exp\{-\lambda d_1(t)\} + exp\{-\lambda d_2(t)\}} \tag{3}$$

In this case, $d_1(t)$ and $d_2(t)$ denote the MSD between the configuration at simulation time t (defined by Cα atoms of TM1, TM2 and TM7) and either the intracellularly closed or intracellularly open conformation, respectively, and λ = 20 nm^{-2}. (Reference open and closed conformations were extracted from MD simulations in different ion-occupancy states.) An additional variable of this type describes the relative opening and closing movements of intracellular portions of TM2 (residues 45-50) and TM8 (residues 232-236), and is defined with three reference states:

$$\xi_{TM2-8}(t) = \frac{exp\{-\lambda\, d_1(t)\} + 2exp\{-\lambda\, d_2(t)\} + 3exp\{-\lambda\, d_3(t)\}}{exp\{-\lambda\, d_1(t)\} + exp\{-\lambda\, d_2(t)\} + exp\{-\lambda\, d_3(t)\}} \tag{4}$$

Here, $d_1(t)$, $d_2(t)$ and $d_3(t)$ are the MSD between the current configuration at time t and three progressively more open reference structures, and λ = 200 nm^{-2}.

The BE-META simulations carried out to explore this alternative reaction-coordinate space included 16 replicas; 15 replicas included Metadynamics potentials on different combinations of reaction coordinates, one replica was unbiased. Additional details are provided in Table S2. Monte-Carlo exchanges were attempted every 5 ps. The total simulation time was 300 ns for each replica; equilibration of the biasing potentials was achieved after approximately 200 ns, and so only the last 100 ns were used for analysis.

Restraining potentials – In the bias-exchange Metadynamics (BE-META) simulations of NCX_Mj with 2 or 3 Na^+ ions bound, we applied restraining potentials on a coordination-number variable

to preclude Na^+ dissociation (even though spontaneous dissociation was not observed in unbiased simulations). The coordination variable was defined as:

$$\xi_C(t) = \sum_k \frac{1-\left(\frac{r_k}{r_0}\right)^8}{1-\left(\frac{r_k}{r_0}\right)^{10}} \quad (5)$$

where r_k is the distance between ions and coordinating oxygen atoms and $r_0 = 0.24$ nm. For this variable we set lower values of $\xi_0 = 4.3$ for Na^+ ions bound to S_{int} and S_{ext} sites (coordinated to 5 oxygens) and $\xi_0 = 4.75$ for the ion bound to site S_{Ca} (coordinated to 6 oxygens; Fig. 2C and Fig. 5C), using a potential of the form $k_C(\xi_C(t) - \xi_0)^4$, applied when $\xi_C(t) < \xi_0$ and for which $k_C = 1{,}000 - 2{,}500$ kJ/mol. The water molecule in the binding site (Fig. 2C and Fig. 5C) was confined with two half harmonic potentials, $k_d(r(t) - r_0)^2$, on distances, $r(t)$, between the water oxygen and the C_γ atoms of N81 and D240 respectively. This potential was set with $k_d = 10{,}000$ kJ/(mol nm^2) and applied when the above mentioned distances are larger than $r_0 = 0.46$ nm.

A similar scheme was used in the BE-METAD simulations with Ca^{2+} bound, but only for the S_{Ca} site. This potential was applied on the coordination variable defined by Eq. 5 in which r_k is the distance between Ca^{2+} and a coordinating oxygen atom, and $r_0 = 0.3$ nm. For this variable we set a lower value of $\xi_0 = 7.4$ using a potential of the form $k_C(\xi_C(t) - \xi_0)^2$, applied when $\xi_C(t) < \xi_0$ and for which $k_C = 400$ kJ/mol. The two water molecules at the S_{mid} site (Fig. 2C) were also confined with a half harmonic potential, $k_d(r(t) - r_0)^4$, on the distances (r) between the C_γ atoms of either N81 or D240 residues with the oxygen atom of the adjacent water molecule. For this potential $r_0 = 0.41$ nm and $k_d = 10{,}000{,}000$ kJ/(mol nm^4). To prevent spurious water penetration and facilitate convergence, we also applied a boundary potential on the coordination variable defined by Eq. 5 in which r_k is the distance between Ca^{2+} and all oxygen atoms of water molecules (excluding the those coordinating Ca^{2+}), and $r_0 = 0.3$ nm. Distances, r_k, beyond 0.5 nm were excluded. For this variable we set an upper value of $\xi_0 = 0.5$ using a potential of the form $k_C(\xi_C(t) - \xi_0)^2$, applied when $\xi_C(t) > \xi_0$ and for which $k_C = 400$ kJ/mol. To prevent unfolding of binding site portions of TM2 and TM7 we also applied harmonic distance restraints ($k_d(r(t) - r_0)^2$, with $r_0 = 0.41$ nm and $k_d = 10{,}000$ kJ/(mol nm^4) between: (1) carbonyl oxygen of A206 and side chain oxygen of S210; (2) carbonyl oxygen of A206 and amide nitrogen of S210; (3) carbonyl oxygen of V205 and amide nitrogen of T209; (4) homologous distance pairs based on inverted symmetry (A47-S51, M46-T50).

<u>Derivation of free-energy landscapes from enhanced-sampling trajectories</u> – To translate the BE-META simulation data into 2D free-energy landscapes, we identified two intuitive structural descriptors of intra- and extracellular opening-closing motions. These descriptors are defined by the number of protein-protein contacts between 1) intracellular portions of TM1-TM2 (residues 17, 20, 21, 24, 25, 42, 43, 46, 47, 48, 50) and TM7-TM8 (residues 211, 212, 213, 215, 216, 219,

220, 231, 232, 235, 236); and 2) extracellular portions of TM6-TM7 (residues 176, 179, 180, 183, 184, 201, 202, 205, 206, 207, 209) and TM2-TM3 (residues 52, 53, 54, 56, 57, 60, 61, 72, 73, 76, 77). Note these sets of residues are in pseudo-symmetrical fragments of the two topological repeats. The number of protein-protein contacts for a given residue selection was defined by:

$$S_C(t) = \sum_{ij} \frac{1 - \left(\frac{r_{ij}(t)}{r_0}\right)^4}{1 - \left(\frac{r_{ij}(t)}{r_0}\right)^8} \quad (6)$$

where r_{ij} denotes the Cα-Cα distance between residue i and j and r_0 , the contact distance, is 0.7 nm.

The free-energy maps shown in Fig. 2A and Fig. 5A reflect a re-weighted histogram as a function of the two descriptors mentioned above. To ensure unbiased sampling, the weight of each simulation frame i was set as:

$$w_i = \frac{1}{N_{\alpha(i)}} \exp\left\{-\frac{F\left(\xi^{\alpha(i)}\right)}{k_\mathrm{B}T}\right\} \quad (7)$$

where $F\left(\xi^{\alpha(i)}\right)$ is the free energy as a function of the reaction coordinates used in the BE-META simulations, $\alpha(i)$ is the bin assigned to frame i and $N_{\alpha(i)}$ is the total number of frames in that bin (19). The free energy, $F\left(\xi^{\alpha(i)}\right)$, was derived using the FCAM method (19). Specifically, with either 3 Na^+ or 1 Ca^{2+} bound, we defined $F\left(\xi^{\alpha(i)}\right)$ as a function of 4 variables, namely ξ_6, ξ_{13}, ξ_{16} and ξ_{17} (Fig. S3), and thus this quantity was deduced from the sampling collected in the BE-META replicas in which those variables were biased (Table S1). The same replicas were used to derive the free energy maps in Fig. 2 based on Eq. 7. The minimum free-energy paths in Fig. 4C and Fig. 4D were also derived using the FCAM software suite (https://github.com/FCAM-NIH/FCAM), again in the space of the 4 reaction coordinates mentioned above.

Maximum-entropy reweighting of simulated ensembles based on HDX-MS data – In its original formulation, the HDXer (20) method assumes that the input datasets of hydrogen deuterium exchange coupled to mass spectrometry (HDX-MS) are corrected for deuterium back-exchange. While this is an ideal situation, not all datasets can be produced with this additional layer of information. For example, the HDX-MS data published for NCX_Mj (24) do not include this correction, to our knowledge. Therefore, it was necessary to develop an alternative strategy that allows for reweighting ensembles on data that have not been corrected for back-exchange levels.

This new scheme assumes that two HDX-MS datasets are available, for which uncorrected deuterium fractions (with values ranging from 0 to 1) of peptide *j* measured at time *t* are denoted as $D_{j,t}^{exp1}$ and $D_{j,t}^{exp2}$ respectively. These datasets refer to experiments carried out under two different biochemical/physiological conditions (e.g., the presence or absence of ligands) or for two different mutants of the same protein. All HDX experiments are assumed to be performed

with the same protocol so that the back-exchange contribution to a given peptide can be reasonably assumed to be the same across the two datasets. In case those datasets correspond to measurements carried out on two protein variants, only identical peptides must be considered for the analysis (i.e., peptides containing sequence variations should be excluded).

A key approximation in this approach is that back-exchange is accounted for by normalization of the deuterated fraction of a given peptide by a factor, c_j, which retains approximately the same value for different timepoints (21, 22) and, as previously mentioned, also across the two different HDX datasets. Provided a fully deuterated control were available, the factor c_j could be experimentally determined using the relation: $c_j = D_{\%}\, n_j/(m_{100} - m_0)$, where m_{100}, m_0 are the centroid masses of the fully-deuterated and non-deuterated peptide respectively, $D_{\%}$ is the D/H fraction in the buffer and n_j is the associated number of exchangeable amide hydrogens. However, in this case we assume that the experimental data are not corrected for back-exchange. Hence, the term c_j is unknown and therefore is set as a free parameter during the reweighting procedure. The implication is that, rather than the absolute experimental deuteration levels, the target of the ensemble reweighting is primarily determined by the variations between the deuterium fractions of the two datasets. Hence, the sensitivity of the reweighting procedure will depend on the magnitude of these variations (in addition to the number of data points).

The proposed scheme involves two concurrent ensemble reweighting calculations, each targeting one of the HDX-MS datasets, and in which the factors c_j are determined at each reweighting step by best fitting to both datasets. The overall likelihood optimization function is given by the sum of the two individual contributions for each dataset (1 and 2):

$$L = \frac{W_{app}^{1}}{k_B T} + \frac{W_{app}^{2}}{k_B T} - \gamma ln P_{err} \tag{8}$$

where $W_{app}^{k=1,2}$ is the apparent work required to reweight the ensemble and P_{err} is the error distribution of the experimental data, including both HDX datasets.
To compute the work requires a model of the exchange for a given frame in the ensemble. As in our previous work, we use the exchange model of Best and Vendruscolo (23):

$$ln P_i^{simk} = \langle \beta_{\mathrm{H}} N_i^H + \beta_{\mathrm{C}} N_i^C \rangle_k \tag{9}$$

in which P_i^{simk} is the predicted protection factor of residue *i* from reweighted ensemble *k*, N_i^H and N_i^C are the number of H-bonds and heavy-atom contacts of the amide group of residue *i*, respectively, and $\langle \dots \rangle_k$ denotes a mean value over the reweighted ensemble *k*. Then, based on the maximum entropy principle, the apparent work can be expressed as (20):

$$W_{app}^{k=1,2}/k_BT = \sum_i \left({}^k\lambda_i^C \langle N_i^C \rangle_k + {}^k\lambda_i^H \langle N_i^H \rangle_k\right) - ln\int exp\left\{-U_{MD}(\mathbf{X}_k)/k_BT + \sum_i \left({}^k\lambda_i^C N_i^C(\mathbf{X}_k) + {}^k\lambda_i^H N_i^H(\mathbf{X}_k)\right)\right\} d\mathbf{X}_k \quad (10)$$

where $N_i^H(\mathbf{X}_k)$ and $N_i^C(\mathbf{X}_k)$ are the number of H-bonds and heavy atom contacts, respectively, for the amide of residue *i*, at a molecular configuration, $\mathbf{X}_k$. We assume that the error distribution, P_{err}, is Gaussian:

$$P_{err} \propto exp\left\{-\sum_k \sum_t \sum_j \left(D_{j,t}^{simk} - c_j D_{j,t}^{expk}\right)^2 / 2\eta_D^2\right\} \quad (11)$$

The normalization parameters, c_j, are determined by minimizing at each step the mean square deviation between simulated and experimental deuteration fractions (i.e. the exponent in Eq. 11), subject to the following constraints (enforced through Lagrange multipliers):

$$c_j \geq 1 \qquad c_j \leq \frac{1}{D_{j,t}^{expk}} \quad (12)$$

The condition $c_j \geq 1$ stems from the consideration that, owing to the back-exchange, $D_{j,t}^{expk}$ is a lower bound of the correct deuteration level. The other boundary constraint ($c_j D_{j,t}^{expk} \leq 1$) instead ensures that the corrected deuteration fraction has an upper boundary of 1. In the case that all boundary constraints are satisfied the expression for the normalization terms is given by:

$$c_j = \frac{\sum_{k=1,2} \sum_t D_{j,t}^{simk} D_{j,t}^{expk}}{\sum_{k=1,2} \sum_t \left(D_{j,t}^{expk}\right)^2} \quad (13)$$

As in our previous work (20), the terms ${}^k\lambda_i^H$ and ${}^k\lambda_i^C$ are iteratively determined in order to minimize the likelihood function of Eq. 8:

$${}^k\lambda_i^{C/H}[n+1] = {}^k\lambda_i^{H/C}[n](1-\epsilon) + \epsilon\gamma\beta_{C/H} \frac{\partial lnP_{err}}{\partial lnP_i^{simk}} \quad (14)$$

where n denotes the iteration step, γ is a parameter that sets the experimental uncertainty and ϵ is a suitably chosen update rate.

Optional optimization of the model parameters – In the previous section we assumed that the model parameters, β_H and β_C, have a fixed value, usually set to that derived by Best and Vendruscolo, which best fit a set of HDX measurements ($\beta_H = 2.0$, $\beta_C = 0.35$) (23). To reduce model bias, we set β_H and β_C as optimizable parameters during reweighting, which are sampled

using a Monte Carlo algorithm and averaged out during reweighting. The parameter sampling stems from choosing an error probability distribution in which β_H and β_C are integrated out:

$$P_{err} = \int exp\left\{-\sum_k \sum_t \sum_j \left(D_{j,t}^{simk} - c_j D_{j,t}^{expk}\right)^2 / 2\eta_D^2\right\} P(\beta_H \beta_C) d\beta_H d\beta_C \qquad (15)$$

where $P(\beta_H \beta_C)$ is the prior error distribution of the model parameters. Applying a prior distribution can be useful for sparse and noisy experimental data, as it restricts the sampling to a reasonable range of values. Here, $P(\beta_H \beta_C)$ was set to mimic the trend of the square residuals as a function of β_H and β_C (Fig. S6B) that was reported by Best and Vendruscolo (23). Specifically, those square residuals describe the discrepancy between predicted and experimental protection factors for a set of proteins. Based on Eq. 15, the update rule for ${}^k\lambda_i^{C/H}$ becomes:

$${}^k\lambda_i^{C/H}[n+1] = {}^k\lambda_i^{H/C}[n](1-\epsilon) + \epsilon\gamma_D \left\langle \beta_{C/H} \frac{\partial ln \acute{P}_{err}(\beta_H, \beta_C)}{\partial ln P_i^{simk}} \right\rangle_{\beta_C,\beta_H} \qquad (16)$$

where $\langle \dots \rangle_{\beta_C,\beta_H}$ denotes an average performed over β_C, β_H parameter space, and

$$\acute{P}_{err}(\beta_H, \beta_C) = exp\left\{-\sum_k \sum_t \sum_j \left(D_{j,t}^{simk} - c_j D_{j,t}^{expk}\right)^2 / 2\eta_D^2\right\} P(\beta_H \beta_C) \qquad (17)$$

The term η_D^2 was set as the minimum value (as a function of β_C, β_H) of the mean square deviation, $MSD = \sum_k \sum_t \sum_j \left(D_{j,t}^{simk} - c_j D_{j,t}^{expk}\right)^2 / N$, before reweighting, in which N is the number of data points. The average in Eq. 16 is performed using Monte Carlo with acceptance probability given by $\acute{P}_{err}(\beta_H, \beta_C)$ in Eq. 17.

_Additional details on the application of the reweighting methodology to NCX_Mj_ – The original HDXer formulation (20) is based on reweighting single protein configurations, which could lead to different weights for very similar structures, for example due to a small structural variation that is sufficient to break a few amide H-bonds. To prevent this, instead of single configurations we reweight free-energy bins. The free-energy of bin α corrected according to the maximum entropy principle is defined as:

$$F_{corr}(\boldsymbol{\xi}^{\alpha}) = F(\boldsymbol{\xi}^{\alpha}) - k_{\mathrm{B}}T \sum_i \left(\lambda_i^H N_{\mathrm{H},i}^{\alpha} + \lambda_i^C N_{\mathrm{C},i}^{\alpha}\right) \qquad (18)$$

where $N_{\mathrm{H},i}^{\alpha}$ and $N_{\mathrm{C},i}^{\alpha}$ are number of amide H-bonds and heavy atoms contacts averaged within bin α. Fig. 3C in the main text was obtained by applying HDXer to the four-dimensional free energy landscape as a function of ξ_6, ξ_{13}, ξ_{16} and ξ_{17} (see above and Fig. S3), based on simulations with 3 Na^+ bound. To prevent a population bias we rescaled the landscape to have 50% probability for both OF and IF states (defined with a 2 kcal/mol cutoff from each minima). We removed intermediate and transition region of the landscape (Fig. 2) as preliminary reweighting trials show

that are not significantly affected by the reweighting. The HDX-MS data for WT and 5L6-8 NCX_Mj used in the HDXer analysis, refer to the measurements reported by Giladi et al. (24), obtained under saturating Na^+ concentration (more consonant with the simulation setup). This data consists of deuteration levels at 15 s, for 12 protein fragments (40-46, 47-59, 53-59, 194-204, 203-214, 205-214, 215-230, 215-237, 219-250, 238-245, 238-250, 259-264), which comprise portions of the α-repeats (TM2, TM7 and TM8, Fig. 3A).

Extraction of conformational ensembles for OF and IF states from free-energy basins – The conformational ensembles considered in comparative analysis of OF, intermediate and IF states were extracted from the BE-META simulations, in reference to the features of the free-energy landscapes. Specifically, the OF state is defined by $\xi_6 \leq 1.3$ and $\xi_{13} \leq 1.55$; the intermediate state by $1.4 < \xi_6 < 1.7$ and $1.35 < \xi_{13} < 1.7$ and $1.4 < \xi_{16} < 1.7$; and the IF state by $\xi_6 > 1.45$ and $\xi_{13} > 1.7$. For the calculation of mean values of a certain descriptor for each ensemble, we included only configurations within 1 kcal/mol from the lowest free-energy configuration in each case. These configurations were selected based on a Metropolis-Monte Carlo acceptance criteria, which accounts for the free energy and thus produces an unbiased sample.

Construction of alternative ion-occupancy states – The states with 2 Na^+ ions bound to NCX_Mj were generated from the states with 3 Na^+ ions bound through a slow alchemical transformation that annihilates the ion bound at either the S_{ext} or the S_{int} site, over a 40-ns MD trajectory. A Cl^- ion in the bulk solution was concurrently annihilated to maintain neutrality. The apo states (i.e., no ions) were constructed from the equilibrated states with 2 Na^+ ions bound by gradually annihilating both ions, again over a 40-ns MD trajectory, together with two Cl^- ions in the bulk solution. The states with 2 H^+ bound were generated from the states with 3 Na^+ bound by gradually annihilating the Na^+ ions from the binding sites, and a Cl^- ion from the bulk, while creating protonated forms of E54 and E213, over a 100-ns MD simulation. Once initial configurations had been obtained, trajectories ranging from 1 to 2 μs were generated for all occupancy states before initiating the enhanced-sampling simulations.

Supporting Tables

Table S1. Gaussian widths (σ), path collective variables smoothing parameters (λ) and combinations of reaction coordinates (Fig. S3) used in each of the BE-META simulations of the alternating-access transition of NCX_Mj with either 3 Na^+ ions or 1 Ca^{2+} ion bound.[a]

Replica	Collective variables biased		σ of biased variables		λ[nm^{-2}] of biased variables	
	1	2	1	2	1	2
1, 17, 33, 49	ξ_1	ξ_2	0.01	0.01	7.5	5
2, 18, 34, 50	ξ_1	ξ_3	0.01	0.013	7.5	7.5
3, 19, 35, 51	ξ_1	ξ_4	0.01	0.027	7.5	57
4, 20, 36, 52	ξ_1	ξ_5	0.01	0.027	7.5	80
5, 21, 37, 53	ξ_1	ξ_6	0.01	0.01	7.5	6
6, 22, 38, 54	ξ_1	ξ_9	0.01	0.013	7.5	5.5
7, 23, 39, 55	ξ_1	ξ_{10}	0.01	0.013	7.5	8
8, 24, 40, 56	ξ_1	ξ_{11}	0.01	0.027	7.5	60
9, 25, 41, 57	ξ_1	ξ_{12}	0.01	0.027	7.5	66
10, 26, 42, 58	ξ_1	ξ_{13}	0.01	0.01	7.5	6.1
11, 27, 43, 59	ξ_7	ξ_8	0.01	0.01	5.6	9.7
12, 28, 44, 60	ξ_{14}	ξ_{15}	0.013	0.013	6.0	10
13, 29, 45, 61	ξ_{16}	ξ_{17}	0.01	0.013	5.3	9.5
14, 30, 46, 62	ξ_1	ξ_{18}	0.01	0.01	7.5	5.9
15, 31, 32, 47, 63, 64	ξ_6	ξ_{13}	0.01	0.01	6.0	6.1
16, 48	unbiased		-		-	

[(a)] Gaussian functions were added to the Metadynamics biasing potential on each variable in 5-ps intervals. In the equilibration stage of Na^+-bound NCX_Mj, we used 32 replicas, all started from the same conformation. To avoid a large initial bias, the strength of the history-dependent potential of Metadynamics was gradually raised during the first 500 ns. Specifically, the Gaussians height was set to 0.25 kJ.mol for the first 360 ns and then gradually incremented to 1 kJ/mol from 360 ns to 500 ns. After 1 μs (for each replica) we duplicated the number of replicas and lowered the Gaussians height to 0.5 kJ/mol for the remaining 200 ns of sampling. For Ca^+-bound NCX_Mj, the Gaussians height was set to 0.5 kJ/mol.

Table S2. Gaussian widths (σ) and temperature bias (ΔT) used in each of the replicas in the bias-exchange well-tempered Metadynamics simulations of opening and occlusion of NCX_Mj[a].

Replica	Collective variables biased	σ(ξ_{int}) [nm]	σ (ξ_{Ca}) [nm]	σ ($\xi_{TM1,2-7}$)	σ (ξ_{TM2-8})	298K+ΔT(K)
1	ξ_{int}, ξ_{Ca}	0.025	0.025	-	-	4470
2	ξ_{int}, ξ_{Ca}	0.025	0.025	-	-	4470
3	ξ_{int}, ξ_{Ca}, ξ_{TM2-8}	0.117	0.117	-	0.233	2214
4	ξ_{int}, ξ_{Ca}, ξ_{TM2-8}	0.100	0.100	-	0.200	1831
5	ξ_{int}, ξ_{Ca}, ξ_{TM2-8}	0.083	0.083	-	0.166	1447
6	ξ_{int}, ξ_{Ca}, ξ_{TM2-8}	0.067	0.067	-	0.133	1064
7	ξ_{int}, ξ_{Ca}, ξ_{TM2-8}	0.050	0.050	-	0.100	681
8	unbiased	-	-	-	-	-
9	$\xi_{TM1,2-7}$, ξ_{TM2-8}	-	-	0.017	0.050	4470
10	$\xi_{TM1,2-7}$, ξ_{TM2-8}	-	-	0.017	0.050	4470
11	ξ_{int}, ξ_{Ca}, $\xi_{TM1,2-7}$	0.117	0.117	0.082	-	2214
12	ξ_{int}, ξ_{Ca}, $\xi_{TM1,2-7}$	0.100	0.100	0.070	-	1831
13	ξ_{int}, ξ_{Ca}, $\xi_{TM1,2-7}$	0.083	0.083	0.058	-	1447
14	ξ_{int}, ξ_{Ca}, $\xi_{TM1,2-7}$	0.067	0.067	0.046	-	1064
15	ξ_{int}, ξ_{Ca}, $\xi_{TM1,2-7}$	0.050	0.050	0.035	-	681
16	$\xi_{TM1,2-7}$, ξ_{TM2-8}	-	-	0.017	0.050	4470

[(a)] Gaussian functions were added to the biasing potential acting on each variable at 5 ps intervals and were initially 2 kJ/mol in height. The height of these Gaussians functions was progressively scaled down as prescribed by the well-tempered Metadynamics method (25). To avoid a large initial bias, the history-dependent potential of well-tempered Metadynamics was reset and restarted at ~60 ns (when several simulation replicas had significantly deviated from the initial protein configuration). Note the specified 'temperature bias' is not the simulation temperature, which was in all cases 298 K. Following a previous study (9), we used this scheme for an efficient exploration of the multidimensional collective-variable space: replicas with larger values of the temperature-bias and Gaussian widths are able to access high free-energy regions, albeit at low spatial resolution. Replicas with smaller values primarily sample low free-energy regions but at high spatial resolution.

Supporting Figures

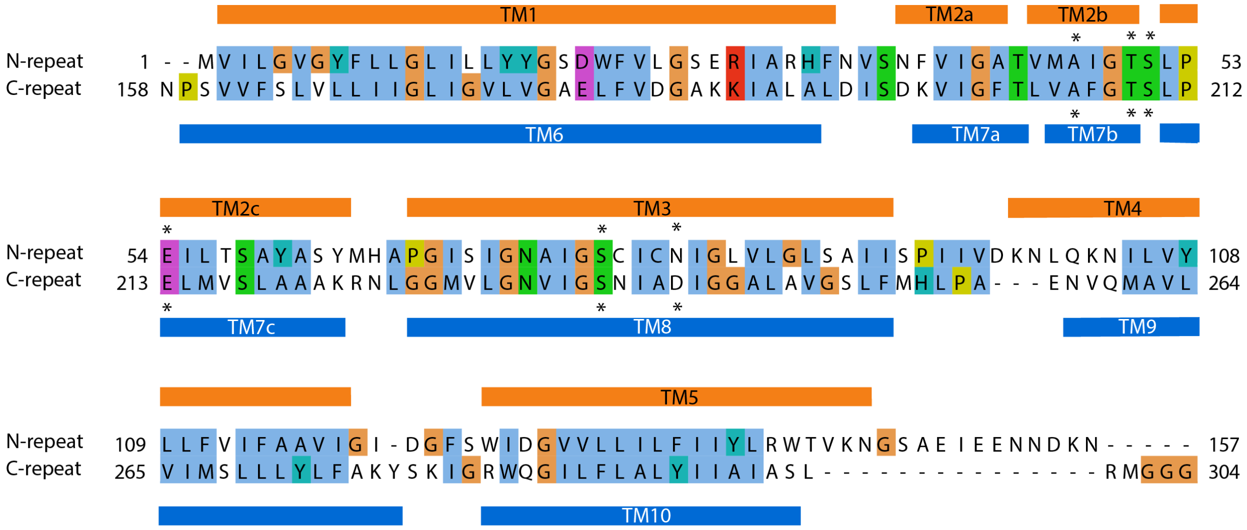

Figure S1. Alignment of the amino-acid sequences of the two inverted topological repeats in NCX_Mj. The sequence identity is 32%. The transmembrane regions in each repeat are indicated and color-coded as in Fig. 1. Residues directly involved in ion-binding are marked with asterisks. The repeat-swap model shown in Fig. 1 was constructed using this alignment.

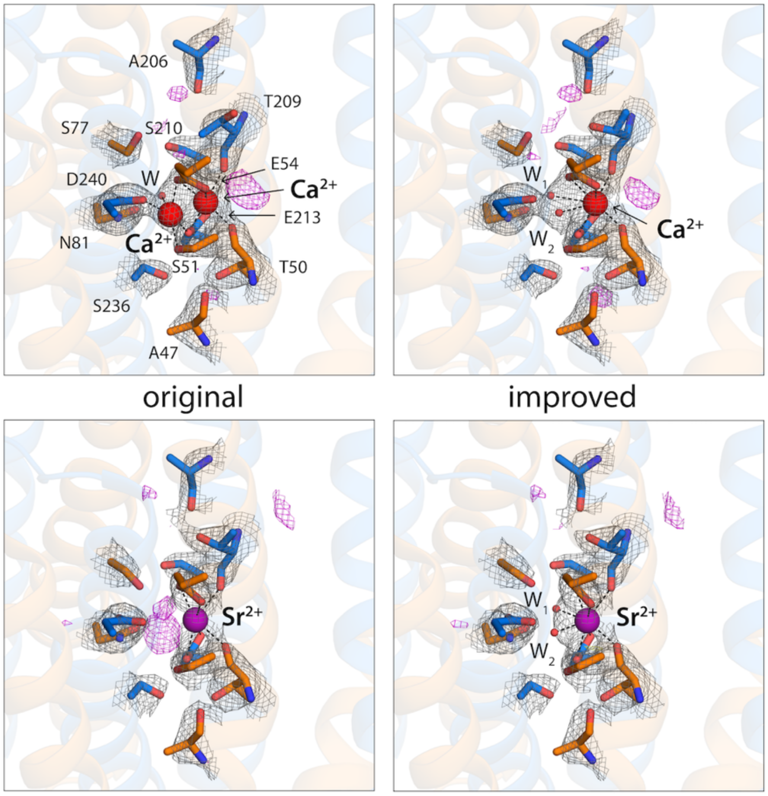

Figure S2. Improved molecular refinement of the Ca^{2+} and Sr^{2+} coordination geometries in outward-facing NCX_Mj based on previously published crystallographic data (9). The original models (PDB entries 5HXR and 5HXS), shown in the left, are contrasted with our proposed interpretation, shown on the right. $2F_o{-}F_c$ maps contoured at 1σ is shown as a grey mesh and $F_o{-}F_c$ omit maps at 3σ is shown in magenta. See Supplementary Methods for further details.

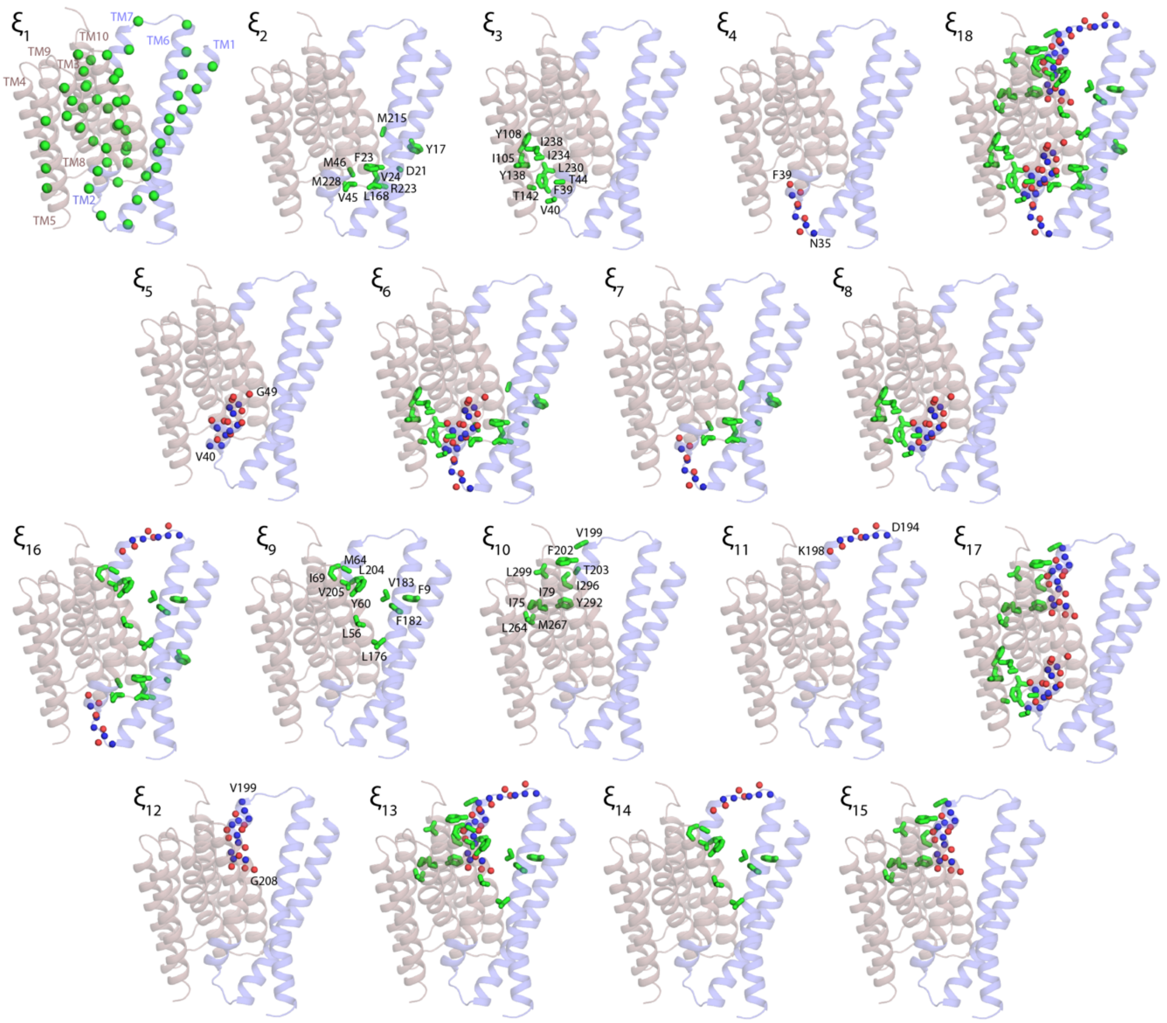

Figure S3. Atom selections used to define the path-collective variables employed in the bias-exchange Metadynamics simulations of the alternating-access transition (see Supplementary Methods). The specific reaction coordinate associated with each selection is indicated (see also Table S1). The selections include Cα atoms (green spheres), backbone N and O atoms (blue and red spheres), and sidechain carbon atoms (green sticks). Note these selections are approximately symmetric, i.e. they include equivalent residues in both topological repeats.

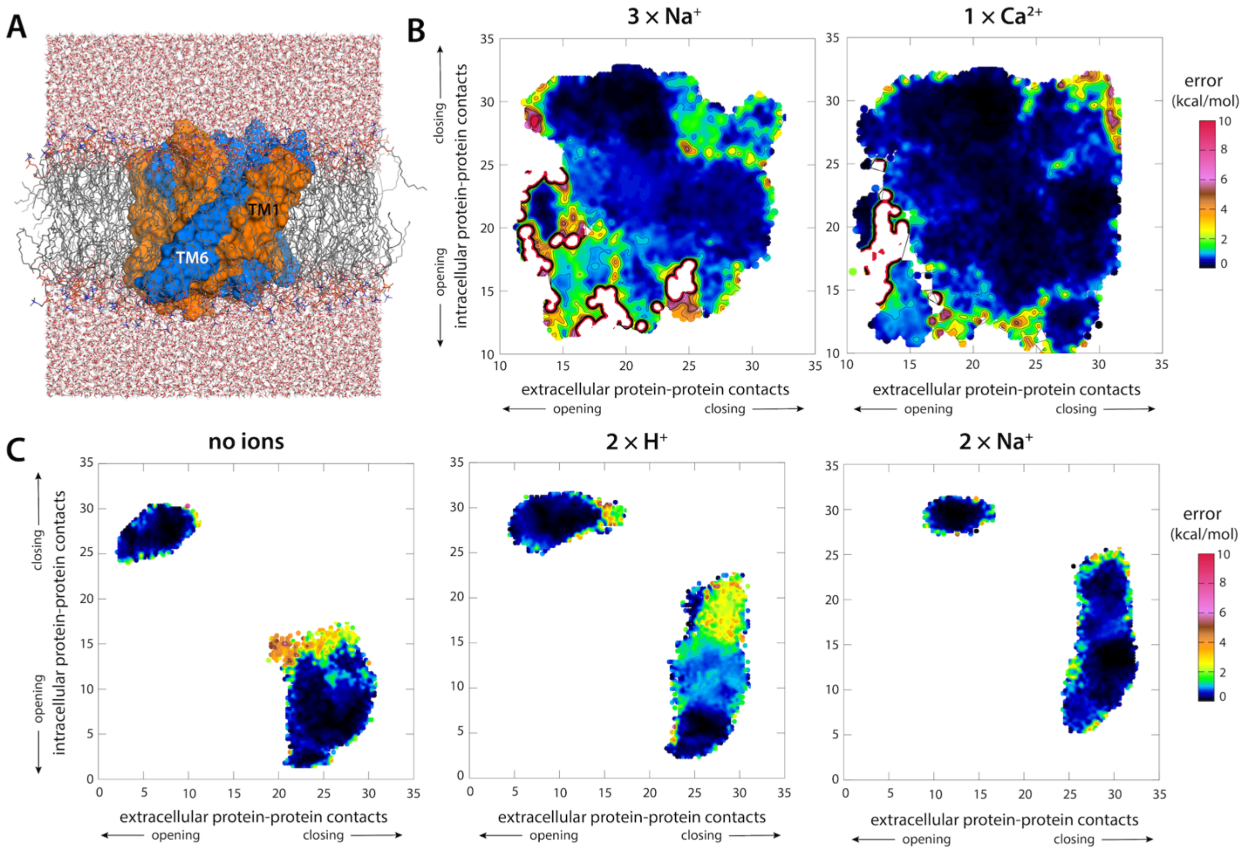

Figure S4. Molecular simulation system and error estimates. (**A**) All-atom simulation system used to calculate the free-energy landscapes shown in Fig. 2 and Fig. 5. The transporter (orange/blue) is embedded in a hydrated POPC lipid bilayer; electrolyte ions are omitted for clarity. (**B**) Estimated error of the free-energy maps shown in Fig. 2, based on block analysis. (**C**) Estimated error of the free-energy maps shown in Fig. 5, also from block analysis.

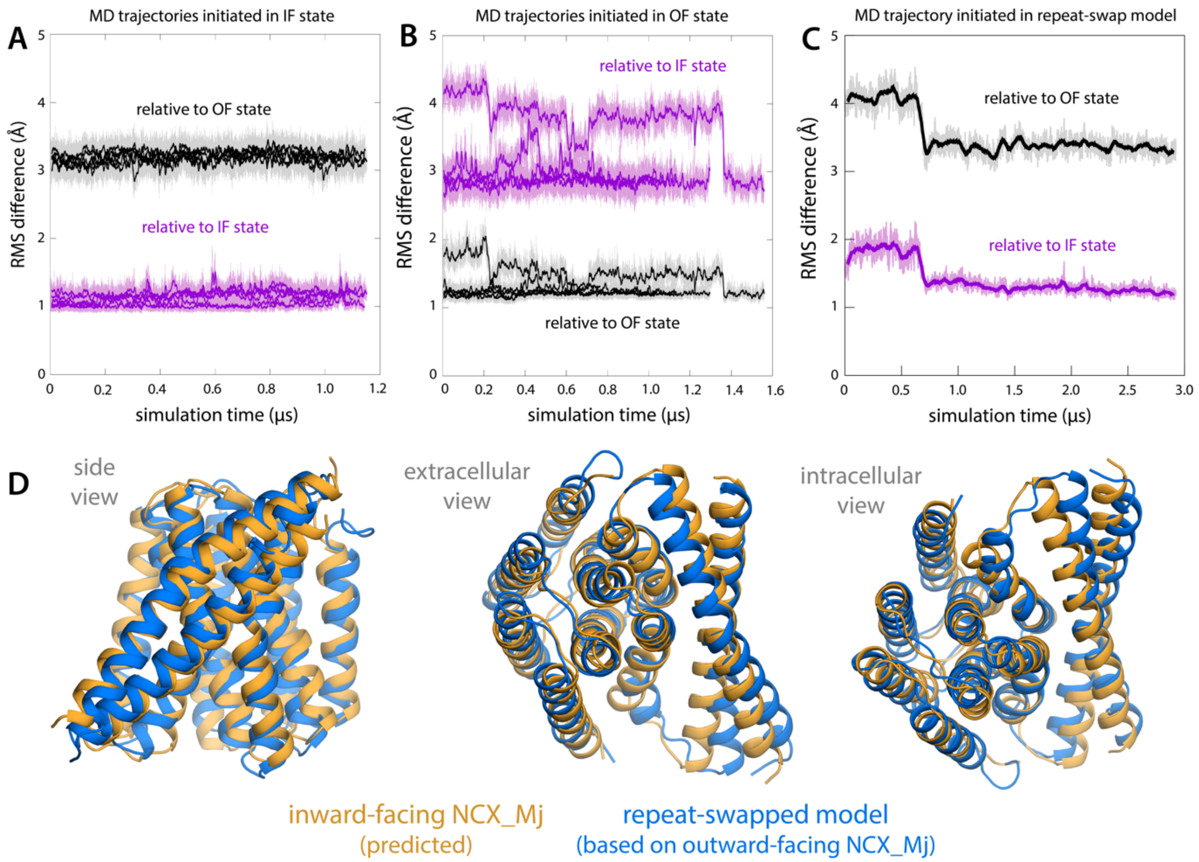

Figure S5. Evaluation of the predicted inward-facing (IF) state of NCX_Mj. (**A**) Conventional MD simulation trajectories initiated in the IF occluded state identified in the free-energy landscape shown in Fig. 2, with 3 Na^+ ions bound. The plot quantifies the average root-mean-square (RMS) difference between the C_α trace at a given simulation time and the C_α traces in an ensemble of structures extracted from each of the two main free-energy basins in that landscape (within 2 kcal/mol of minimum), i.e. the original IF state, and the outward-facing (OF) occluded state. Unstructured loop regions were omitted in this analysis. (**B**) Same as (A), for trajectories initiated instead in the OF occluded state. (**C**) Same as (A), for a trajectory initiated in the repeat-swap model shown in Fig. 1. (**D**) Overlay of the repeat-swap model shown in Fig. 1 and the predicted IF occluded state identified in the free-energy landscape shown in Fig. 2, as noted. The RMS difference (backbone only) is about 2 Å.

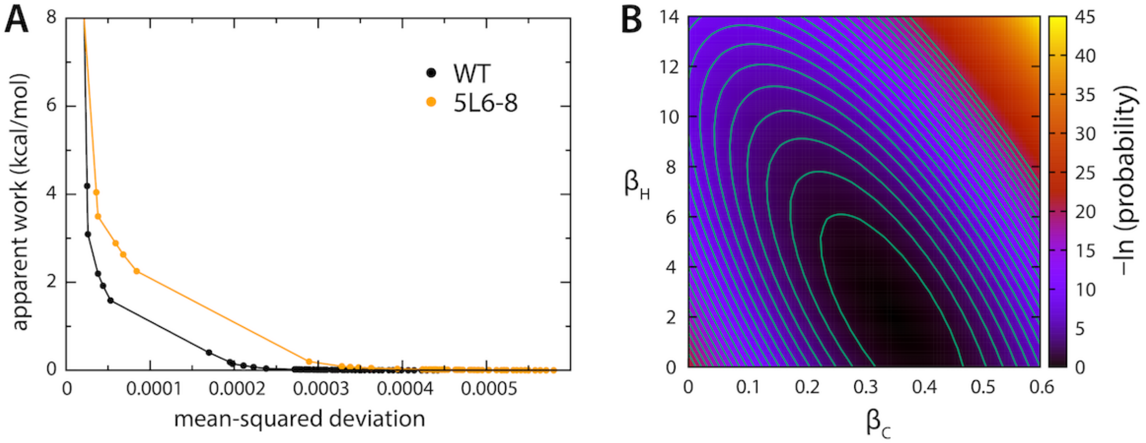

Figure S6. Evaluation of the predicted conformational ensembles for OF and IF states, in reference to HDX-MS data for two forms of NCX_Mj with different propensities for the OF and IF states. See also Fig. 3C. (**A**) The plot quantifies the degree to which a conformational ensemble that initially consists of equally weighted populations OF and IF structures (extracted from simulation) must be re-reweighted to produce optimal agreement with the experimental data obtained for each protein construct (see Supplementary Methods). The degree of reweighting is quantified by an 'apparent work', and the agreement with experiment by the mean-squared deviation between calculated and measured deuteration fractions for a collection of protein fragments. (**B**) Prior probability distribution of the model parameters used to predict hydrogen-deuterium exchange protection factors based on the forward model of Best and Vendruscolo (23).

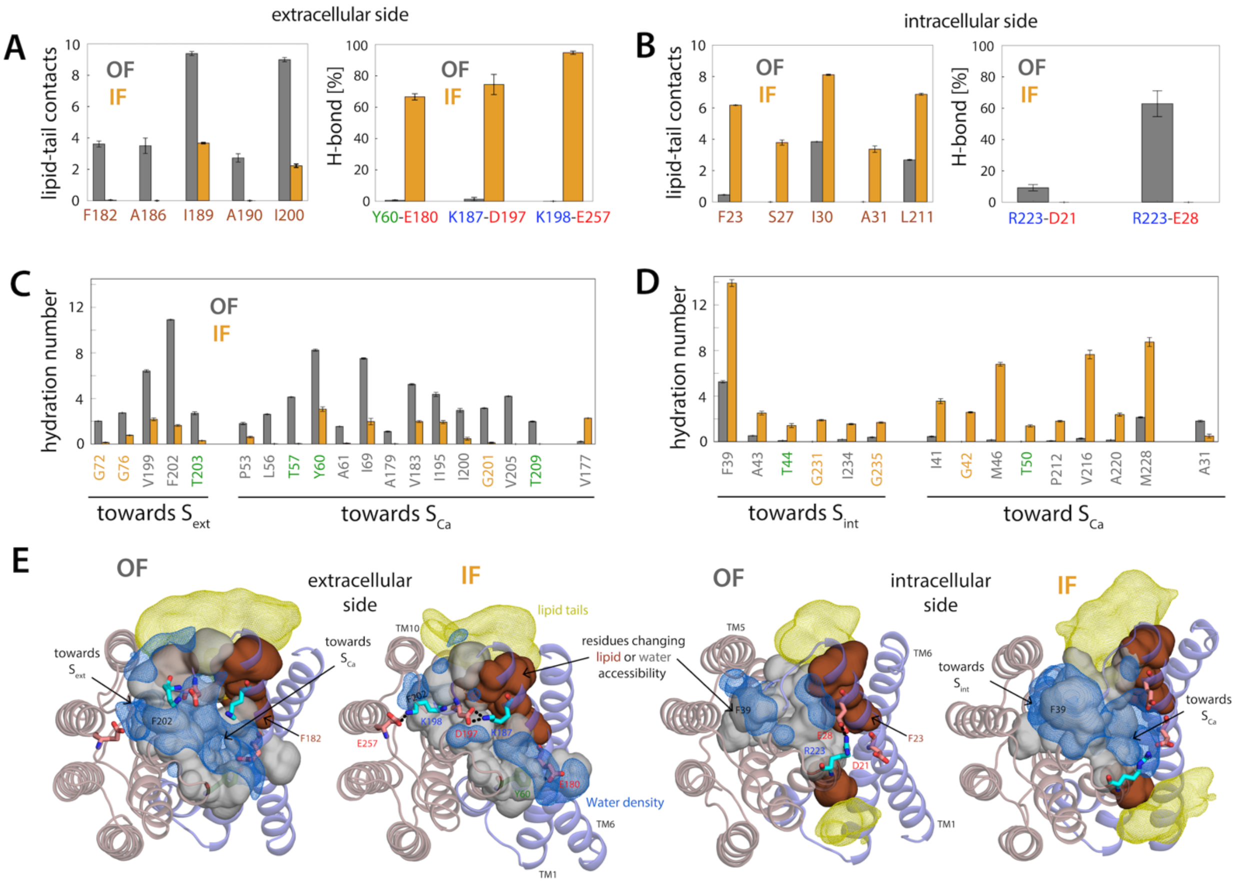

Figure S7. Changing interaction patterns in the alternating-access mechanism of NCX_Mj. Reported quantities are calculated for ensembles of either OF or IF structures, with 3 Na^{+} ions bound, extracted from the free-energy basins revealed in the landscapes shown in Fig. 2. (**A, B**) The charts enumerate the set of residues in the extracellular or intracellular side of the protein, respectively, that show a significant change in hydrophobic lipid-tail contacts or protein-protein H-bonding and salt-bridges when OF and IF stats are compared. (**C, D**) The charts enumerate the set of residues in the extracellular or intracellular side of the protein, respectively, that show a change in hydration number of 2-fold or more; the hydration number was defined using a cutoff distance of 4.2 Å. (**E**) Representative structures of the OF and IF ensembles illustrating the changes in interaction patterns quantified in panels (A,B) and (C,D). The residues that exhibit changes in water accessibility and lipid contacts are shown as grey and brown volumes, respectively, with water and lipid molecules in their vicinity shown with density isosurfaces (blue and yellow mesh, respectively). Residues that exhibit changes in protein-protein H-bonding and salt bridges are shown as sticks. The number of lipid-tail and H-bond contacts between two groups of atoms in a given simulation snapshot was calculated using distance cut-off values of 5.2 and 3.2 Å, respectively. Hydrogen atoms were excluded from this analysis.

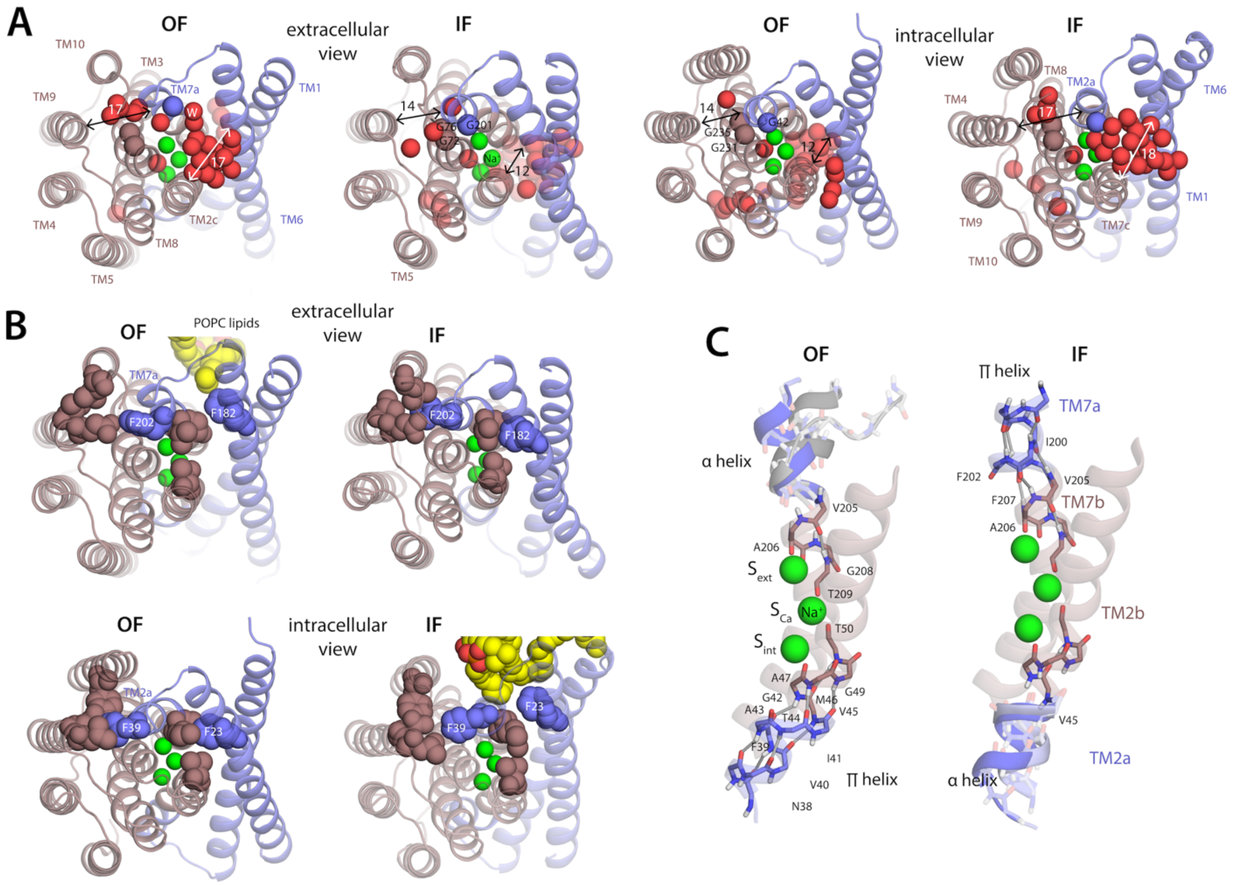

Figure S8. Changing interaction patterns in the alternating-access mechanism of NCX_Mj. (**A**) Extracellular and intracellular views of representative OF and IF conformations, with 3 Na^+ ions bound (green spheres), highlighting water molecules within 10 Å of the ion-binding sites (red spheres). The approximate distances between TM helices involved in the opening and closing of intra- and extracellular pathways are indicated in Å. (**B**) Same views as panel (A), showing phenylalanine sidechains (spheres) that occupy or become released from hydrophobic pockets during the alternating access transition (F182 and F202 on the extracellular side, and F23 and F39 on the intracellular side). Protein residues that are in contact with those phenylalanine sidechains when buried in a hydrophobic pocket are also highlighted, as are lipid molecules in contact with either F23 or F182 when disengaged (yellow). (**C**) Close-up of the backbone of N-terminal portions of TM2 (TM2a-TM2b) and TM7 (TM7a-TM7b) in the OF and IF state, highlighting selected intramolecular H-bonds (gray lines).

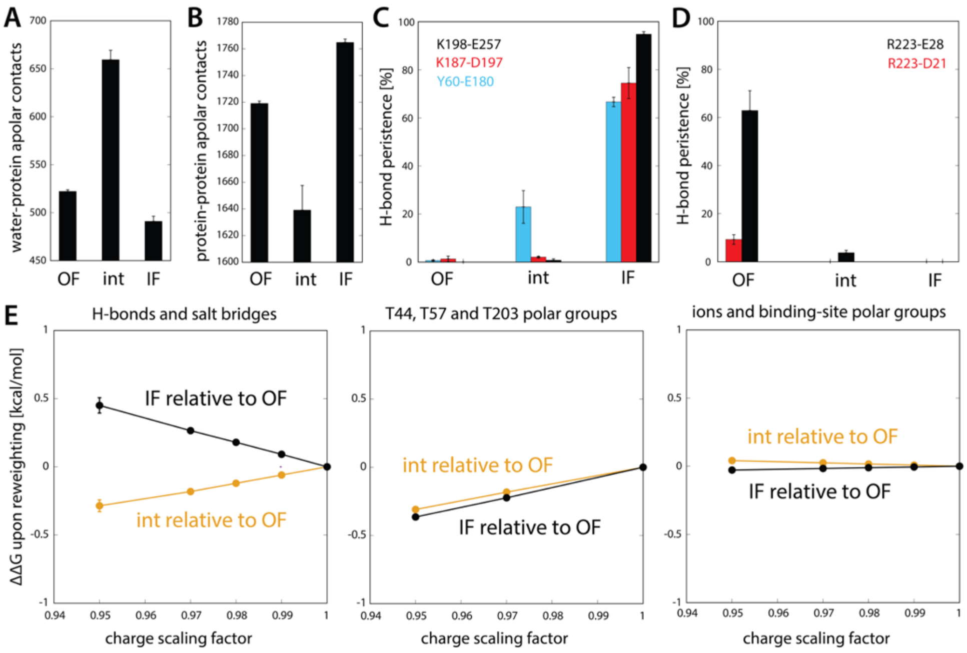

Figure S9. Global changes in hydrophobic, H-bond and electrostatic interactions in the alternating-access mechanism of NCX_Mj. Reported quantities are calculated for ensembles of either OF or IF structures, with 3 Na^+ ions bound, extracted from the free-energy basins revealed in the landscapes shown in Fig. 2. Data is also shown for an ensemble of intermediate configurations. (**A**) Number of contacts between water and protein apolar groups in each state, calculated as in Fig. S7, for r_0 = 4.2 Å .The residues selected are those that change significantly their accessibility to water during the alternating-access transition, namely: 24, 31, 38, 39, 41, 42, 43, 44, 46, 50, 53, 56, 57, 60, 61, 66, 68, 69, 72, 76, 138, 177, 179, 183, 195, 198, 199, 200, 201, 202, 203, 205, 209, 212, 216, 220, 225, 228, 231, 234, 235, and 260. (**B**) Number of contacts between the apolar portion of the previously mentioned protein residues and the apolar portion of all other protein residues, for each state. The number of contacts between two groups of atoms indexed by i and j, for a given simulation snapshot at time t, was calculated as $S(t) = \sum_{ij}[1 - (r_{ij}(t)/r_0)]^8/[1 - (r_{ij}(t)/r_0)]^{16}$, where r_{ij} is the distance between any pair of atoms and r_0 is a constant that depends on the type of interaction; the values used for water-protein and protein-protein contacts were 4.2 and 5.2 Å, respectively. (**C**) Persistence of H-bonding interactions between residues on the extracellular side of the protein, for each state; only interactions that change significantly during the transition are examined. (**D**) Same as (C), for residues on the intracellular side. (**E**) Analysis of the contribution of electrostatic interactions to the free-energy difference between the OF state and either the IF or the intermediate state, evaluated after scaling down the charges of selected atom groups (via a multiplication factor). To quantify the contribution of H-bonds and salt bridges, we scaled down the atomic charges in the polar groups of residues D21, E28, Y60, E180, K187, D197, K198, R223 and E257 (specifically, to the atoms in each of the 'charge groups' bearing either a neutral or finite integer charge e.g. the $-CH_2-COO^-$ group of glutamate or aspartate). To assess the contribution of polar residues changing accessibility during the transition, we scaled down the charges of the polar groups of T44, T57 and T203. The contribution of the binding site was evaluated by scaling down the charges of the polar groups of residues coordinating the bound Na^+ and water molecules, as well the ions and water molecules. To estimate the change in these free-energy differences, we resampled the BE-META trajectories (with 3 Na^+ bound, for the replicas with bias on ξ_6, ξ_{13}, ξ_{16} or ξ_{17}; see Table S1), introducing a reweighing factor for each configuration, i, given by $W_i = exp\ \{-(U_i^M - U_i)/k_B T\}$, where the term $(U_i^M - U_i)$ is the difference between the potential-energy functions with modified and conventional charges, respectively.

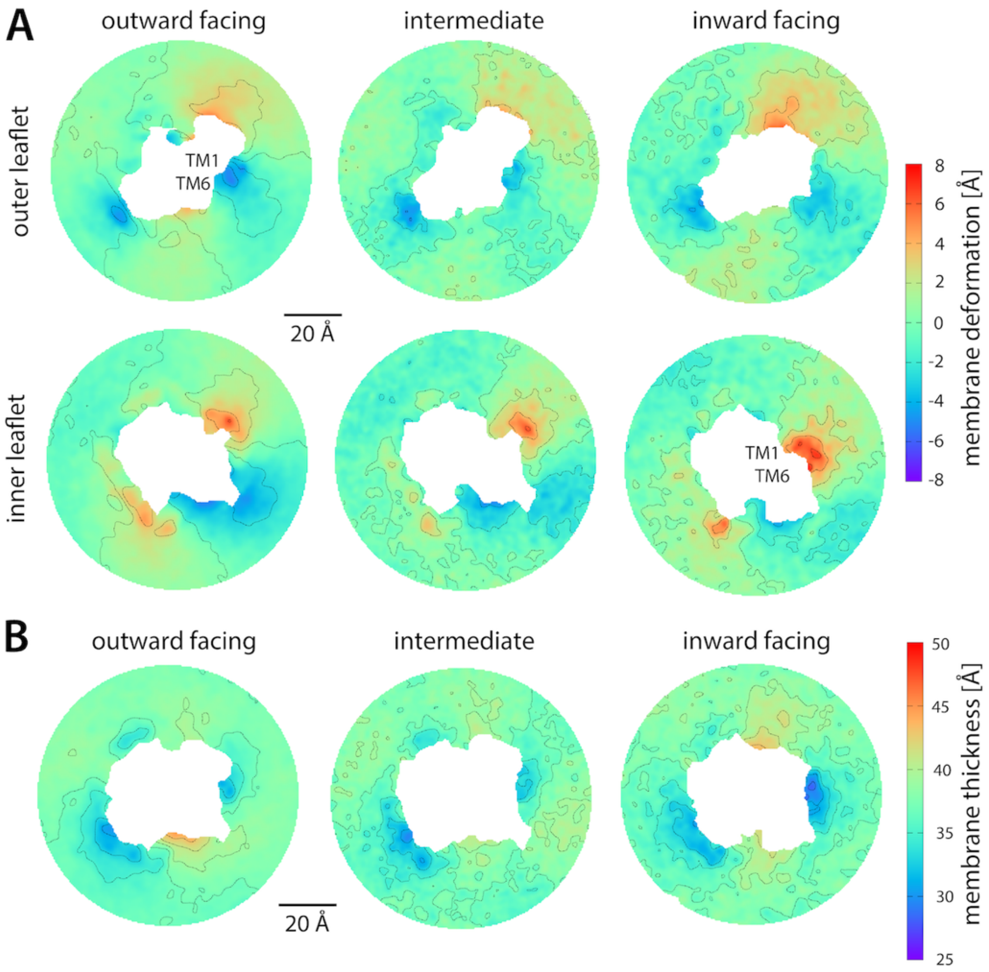

Figure S10. Changes in membrane shape and thickness during the alternating access transition. (**A**) Deformation of each of the lipid bilayer leaflets relative to a flat shape, for the OF, intermediate and IF states. To quantify this deformation, we evaluated the mean Z-coordinate of the ester layer across the XY plane relative to the mean Z-coordinate of both layers, in the bulk region (at a distance larger that 45 Å from the ion-binding sites center, where the membrane is approximately flat). (**B**) Hydrophobic thickness of the lipid bilayer in the OF, intermediate and IF states. The thickness was evaluated as the difference between the mean Z-coordinates of the outer and inner ester layers. Contours are shown in intervals of 2 Å. All maps were calculated after first aligning the protein onto a reference frame, using the portion of the structure that is invariant when OF and IF states are compared (Fig. 4AB). To prevent tilting of the membrane during this process, this alignment was two-dimensional (i.e. only X and Y components of the atomic coordinates are considered). After structural alignment we calculated for each state a three-dimensional occupancy map for the ester oxygen atoms of all POPC lipids in either the outer or the inner leaflet, using the Volmap tool of VMD. The mean Z-coordinate was calculated as the weighted average of the Z-coordinate for each value of X and Y, with a weight provided by the occupancy at that position. Values of X and Y featuring less than 1% of the overall occupancy are excluded (and correspond to the region occupied by the protein in the center of each map).

Supplementary References

1. A. Vergara-Jaque, C. Fenollar-Ferrer, D. Kaufmann, L. R. Forrest, Repeat-swap homology modeling of secondary active transporters: updated protocol and prediction of elevator-type mechanisms. *Front Pharmacol* **6**, 183 (2015).
2. A. Sali, J. P. Overington, Derivation of rules for comparative protein modeling from a database of protein structure alignments. *Protein Sci* **3**, 1582-1596 (1994).
3. J. Liao *et al.*, Structural insight into the ion-exchange mechanism of the sodium/calcium exchanger. *Science* **335**, 686-690 (2012).
4. G. G. Krivov, M. V. Shapovalov, R. L. Dunbrack, Jr., Improved prediction of protein side-chain conformations with SCWRL4. *Proteins* **77**, 778-795 (2009).
5. A. D. MacKerell *et al.*, All-atom empirical potential for molecular modeling and dynamics studies of proteins. *J Phys Chem B* **102**, 3586-3616 (1998).
6. A. D. Mackerell, Jr., M. Feig, C. L. Brooks, 3rd, Extending the treatment of backbone energetics in protein force fields: limitations of gas-phase quantum mechanics in reproducing protein conformational distributions in molecular dynamics simulations. *J Comput Chem* **25**, 1400-1415 (2004).
7. R. Staritzbichler, C. Anselmi, L. R. Forrest, J. D. Faraldo-Gomez, GRIFFIN: A versatile methodology for optimization of protein-lipid interfaces for membrane protein simulations. *J Chem Theory Comput* **7**, 1167-1176 (2011).
8. J. C. Phillips *et al.*, Scalable molecular dynamics on CPU and GPU architectures with NAMD. *J Chem Phys* **153**, 044130 (2020).
9. J. Liao *et al.*, Mechanism of extracellular ion exchange and binding-site occlusion in a sodium/calcium exchanger. *Nat Struct Mol Biol* **23**, 590-599 (2016).
10. P. Emsley, K. Cowtan, Coot: model-building tools for molecular graphics. *Acta Crystallogr D Biol Crystallogr* **60**, 2126-2132 (2004).
11. P. D. Adams *et al.*, PHENIX: a comprehensive Python-based system for macromolecular structure solution. *Acta Crystallogr D Biol Crystallogr* **66**, 213-221 (2010).
12. M. Iwaki *et al.*, Structure-affinity insights into the Na^+ and Ca^{2+} interactions with multiple sites of a sodium-calcium exchanger. *The FEBS Journal* (2020).
13. M. Barthmes, J. Liao, Y. Jiang, A. Bruggemann, C. Wahl-Schott, Electrophysiological characterization of the archaeal transporter NCX_Mj using solid supported membrane technology. *J Gen Physiol* **147**, 485-496 (2016).
14. F. Marinelli *et al.*, Sodium recognition by the Na^+/Ca^{2+} exchanger in the outward-facing conformation. *Proc Natl Acad Sci U S A* **111**, E5354-5362 (2014).
15. J. D. Faraldo-Gomez, B. Roux, On the importance of a funneled energy landscape for the assembly and regulation of multidomain Src tyrosine kinases. *Proc Natl Acad Sci USA* **104**, 13643-13648 (2007).
16. F. Marinelli *et al.*, Evidence for an allosteric mechanism of substrate release from membrane-transporter accessory binding proteins. *Proc Natl Acad Sci USA* **108**, E1285-E1292 (2011).
17. C. Corbi-Verge *et al.*, Two-state dynamics of the SH3-SH2 tandem of Abl kinase and the allosteric role of the N-cap. *Proc Natl Acad Sci USA* **110**, E3372-E3380 (2013).
18. F. Marinelli, Following Easy Slope Paths on a Free Energy Landscape: The Case Study of the Trp-Cage Folding Mechanism. *Biophys J* **105**, 1236-1247 (2013).
19. F. Marinelli, J. D. Faraldo-Gomez, Force-Correction Analysis Method for derivation of multidimensional free-energy landscapes from adaptively biased replica simulations. *J Chem Theory Comput* **17**, 6775-6788 (2021).

20. R. T. Bradshaw, F. Marinelli, J. D. Faraldo-Gomez, L. R. Forrest, Interpretation of HDX data by maximum-entropy reweighting of simulated structural ensembles. *Biophys J* **118**, 1649-1664 (2020).
21. Z. Zhang, D. L. Smith, Determination of amide hydrogen exchange by mass spectrometry: a new tool for protein structure elucidation. *Protein Sci* **2**, 522-531 (1993).
22. G. R. Masson *et al.*, Recommendations for performing, interpreting and reporting hydrogen deuterium exchange mass spectrometry (HDX-MS) experiments. *Nat Methods* **16**, 595-602 (2019).
23. R. B. Best, M. Vendruscolo, Structural interpretation of hydrogen exchange protection factors in proteins: characterization of the native state fluctuations of CI2. *Structure* **14**, 97-106 (2006).
24. M. Giladi *et al.*, Dynamic distinctions in the Na^{+}/Ca^{2+} exchanger adopting the inward- and outward-facing conformational states. *J Biol Chem* **292**, 12311-12323 (2017).
25. A. Barducci, G. Bussi, M. Parrinello, Well-tempered metadynamics: a smoothly converging and tunable free-energy method. *Phys Rev Lett* **100**, 020603 (2008).
